# Bioavailability Assessment of an Iron Formulation Using Differentiated Human Intestinal Caco-2 Cells

**DOI:** 10.3390/foods12163016

**Published:** 2023-08-11

**Authors:** Melissa Fanzaga, Carlotta Bollati, Giulia Ranaldi, Sabrina Sucato, Silvia Fustinoni, Gabriella Roda, Carmen Lammi

**Affiliations:** 1 Department of Pharmaceutical Sciences, University of Milan, Via Mangiagalli 25, 20133 Milan, Italy; melissa.fanzaga@unimi.it (M.F.); carlotta.bollati@unimi.it (C.B.); gabriella.roda@unimi.it (G.R.); 2 CREA-Research Centre for Food and Nutrition, Via Ardeatina, 546, 00178 Rome, Italy; giulia.ranaldi@crea.gov.it; 3 Department of Clinical Sciences and Community Health, University of Milan, 20122 Milan, Italy; sabrina.sucato@unimi.it (S.S.); silvia.fustinoni@unimi.it (S.F.); 4 IRCCS Ca’ Granda Foundation Maggiore Policlinico Hospital, 20122 Milan, Italy

**Keywords:** absorption, Caco-2 cells, food iron, dietary supplement, TEER, trans-epithelial transport

## Abstract

In recent years, there has been growing interest in exploring alternative and innovative delivery systems to improve the efficacy of iron supplements, satisfying iron needs and lowering side effects. To address this issue, this study aimed at demonstrating the advantages of Ferro Supremo formulation (composed of encapsulated iron, vitamins, and micronutrients), in terms of capacity to improve iron intestinal absorption, in comparison with standard FeSO_4_. Hence, differentiated Caco-2 cells have been used for assessing the in vitro bioavailability and safety of FS and FeSO_4_. MTT experiments demonstrated that both FS and FeSO_4_ are not able to impair the viability of Caco-2 cells. Furthermore, the quantitative and qualitative analysis, conducted by atomic absorption spectrometry and fluorescence determinations, revealed that FS can enter, accumulate in the cytoplasm, and be transported by intestinal cells four times more efficiently than FeSO_4_. Our findings indicate that this formulation can be considered a valuable and efficiently good choice as food supplements for improving iron deficiency.

## 1. Introduction

Iron is an essential mineral needed for human life, greatly available in the earth’s crust [1]. It is a vital component for many body metabolic functions, such as hemoglobin synthesis, oxygen transport, DNA synthesis, and electron transport [2]. In 2015, EFSA reported a Scientific Opinion on Dietary Reference Values for iron, stating that the Average Requirement (AR), considering predicted iron absorption values of 16% for men and 18% for women, is 6 mg/day for men and postmenopausal women, resulting to be higher for premenopausal women (16 mg/day) and for children, in accordance with their period of growth [3].

The majority of the body's iron is found in the hemoglobin of erythrocytes in the form of heme; around 10–20% is stored in the liver in the form of ferritin and can be mobilized when needed; around 4% is bound to muscle myoglobin, and the remaining percentage is found in several enzymes, in particular, the ones involved in oxidative metabolism [4]. The average body iron content is 3.5–4 g for women and 4–5 g in men, [1] while the average dietary iron absorbed through the intestinal system is around 1–2 mg each day, compensating for the daily iron loss caused by the physiologic exfoliation of epithelial surfaces or bleeding [5]. Dietary iron exists as heme and non-heme iron. Heme iron is abundant in meat and fish in the form of hemoglobin and myoglobin, and it’s well absorbed by enterocytes after its release by the action of gastric and intestinal proteases, thanks to the presence of transporters on the intestinal microvilli surface [6]. Non-heme iron mainly derives from plants and its absorption is much lower, as well as being affected by other food components as polyphenols and tannins [7]. Given the biological importance of this essential trace element and the possible toxicity caused by the generation of reactive oxygen species (ROS) formed in case of excess iron, a fine modulation of its uptake, transport, storage, and metabolism is crucial to keep iron homeostasis in accordance with the body needs, seeing key regulators as hepcidin playing a fundamental role in these biological processes [1,8]. As a matter of fact, iron exhibits a U-shaped risk curve, where both its deficiency or overload can cause problems for the human body: its inadequate intake leads to several metabolic abnormalities, whilst an excessive iron dose is known to potentially react in inorganic reactions, damaging lipid membranes and organelles, besides promoting gut pathogens proliferation [9,10]. Iron deficiency anemia represents a significant public health problem, and it is the most common form of anemia, whose major causes are linked to blood loss (menstrual periods, gastrointestinal bleeding), chronic kidney disease (CKD), inflammatory diseases, malabsorption, as well as pregnancy and iron-poor diets [11].

One way to prevent this problem could be the fortification of foods [12] or iron supplementation [9]. Conventional iron supplementation is usually recommended as first-line therapy, and it is widely used thanks to its low cost and effectiveness, seeing FeSO_4_ as the most prescribed oral iron among the available types of iron supplements (200 mg dose to treat iron deficiency anemia). Three ferrous iron salts, sulphate, fumarate, and gluconate, are the standard recommended supplements for iron deficiency anaemia (IDA), generally all provided with vitamin C in their formulations [13]. Indeed, it is well established that the presence of vitamin C can increase iron absorption, by creating a more acidic environment in the stomach and preventing the oxidization of ferrous iron to ferric iron [14]. The shortcomings of these supplements, however, concern the high frequency of gastrointestinal side effects, which lead to inadequate treatment adherence, as well as their low bioavailability [15,16,17].

As a result, in recent years there has been growing interest in exploring alternative delivery systems to improve both the safety and efficacy of iron supplements, avoiding soluble iron to induce negative side-effects, while still remaining bioavailable and being more palatable [18]. Liposomes have been often used as versatile carriers to successfully encapsulate several food ingredients as vitamins and minerals, enhancing the efficiency of nutrients absorption, mainly because they can enter the body easily through membrane fusion or phagocytosis, avoiding the protein-mediated transport pathways, and directly enter the blood circulation after crossing the intestinal epithelium [19,20]. Hence, this study focused on studying the bioavailability and safety of Ferro Supremo (FS), a formulation of liposomal iron, whose composition includes vitamin C, copper, and riboflavin, as a valid alternative of FeSO_4_ in food supplements products. Indeed, we aimed at demonstrating the advantages of this specific formulation composed of encapsulated iron, in addition to vitamins and micronutrients, in terms of capacity to improve iron intestinal absorption. For this purpose, differentiated human intestinal Caco-2 cells, a worldwide cell line recognized to simulate the intestinal barrier, have been used for the intestinal absorption and safety investigation of FS, using in parallel FeSO_4_ as a reference compound.

## 2. Materials and Methods

### 2.1. Chemicals

Dulbecco’s modified Eagle’s medium (DMEM), stable L-glutamine, fetal bovine serum (FBS), phosphate buffered saline (PBS), penicillin/streptomycin, nonessential amino-acid, and 96-well plates were purchased from Euroclone (Milan, Italy). MTT [3-(4,5-dimethylthiazol-2-yl)-2,5-diphenyltetrazolium bromide, Merocyanine 540, NaCl, KCl, CaCl_2_, MgCl_2_, glucose, morpholinoethane sulfonic acid, *N*-2-hydroxyethylpiperazine-*N*-4-butanesulfonic acid, 40,6-diamidino-2-phenylindole dihydrochloride (DAPI), FITC-conjugated phalloidin, paraformaldehyde, and vitamin C (Ascorbic acid) were bought from Sigma-Aldrich (St. Louis, MO, USA), the pro-long antifade mounting medium was from ThermoFisher. The liposomial iron (FS), whose composition is shown in Table 1 was provided by Natural Poin S.r.l. (Milan, Italy), and FeSO_4_ were commercially available.

### 2.2. Merocyanine 540 (MC540) Labeling

The dye stock solution of MC540 50 μM was prepared before use, in distilled H_2_O, and kept in the dark. FS (50 mg) was incubated with MC540 solution in a final volume of 1 mL, kept in the dark, and constantly shaken for 1 h at RT. After incubation, it was centrifuged at 6000 rpm for 1 min, the supernatant was removed and the pellet was washed twice with distilled H_2_O. Finally, 1 mL of distilled H_2_O was added to the pellet in order to obtain labeled FS at a final concentration of 50 mg/mL.

### 2.3. Caco-2 Cell Culture and Differentiation

Human intestinal Caco-2 cells, obtained from INSERM (Paris, France), were routinely cultured in DMEM containing 25 mM of glucose, 3.7 g/L of NaHCO_3_, 4 mM of stable L-glutamine, 1% nonessential amino acids, 100 U/L of penicillin, and 100 μg/L of streptomycin (complete medium), supplemented with 10% heat-inactivated fetal bovine serum (FBS; Hyclone Laboratories, Logan, UT, USA). For differentiation, they were seeded on polycarbonate filters, 12 mm diameter, 0.4 µm pore diameter (Transwell, Corning Inc., Lowell, MA, USA) at a 3.5 × 10^5^ cells/cm^2^ density in complete medium supplemented with 10% FBS in both AP and BL compartments for 2 days to allow the formation of a confluent cell monolayer, cell were then supplemented with 10% FBS only in BL compartment, and allowed to differentiate for 15 days with regular medium changes three times weekly [21].

### 2.4. 3-(4,5-Dimethylthiazol-2-yl)-2,5-diphenyltetrazolium Bromide (MTT) Assay

The MTT experiments were conducted on human intestinal Caco-2 cells. Briefly, a total of 3 × 10^4^ cells/well were seeded in 96-well plates and, after 24 h, treated with FeSO_4_ (in the presence of vitamin C) and/or FS from 0.1 to 1.0 mg/mL, or vehicle, in complete growth media for 48 h at 37 °C under 5% CO_2_ atmosphere. Subsequently, the treatment was aspirated, and 100 µL/well of 3-(4,5-dimethylthiazol-2-yl)-2,5-diphenyltetrazolium bromide (MTT) filtered solution was added. After 2 h of incubation at 37 °C under a 5% CO_2_ atmosphere, 0.5 mg/mL solution was aspirated and 100 µL/well of the lysis buffer (8 mM HCl + 0.5% NP-40 in DMSO) was added. After 10 min of slow shaking, the absorbance at 575 nm was read on the Synergy H1 fluorescence plate reader (Biotek, Bad Friedrichshall, Germany).

### 2.5. Cell Monolayers Integrity Evaluation

The trans-epithelial electrical resistance (TEER) of differentiated Caco-2 cells was measured at 37 °C using the voltmeter apparatus Millicell (Millipore Co., Billerica, MA, USA), immediately before, at 30 min, and at the end of the transport experiments (60 min).

### 2.6. Trans-Epithelial Transport Experiments

Before transport experiments cells were maintained 16 h in FBS-free culture media both in AP and BL compartments. Prior to experiments, cell monolayer integrity and differentiation were checked by TEER measurement as described in detail above. Samples of trans-epithelial passage were assayed in differentiated Caco-2 cells in transport buffer solution (137 mM NaCl, 5.36 mM KCl, 1.26 mM CaCl_2_, and 1.1 mM MgCl_2_, 5.5 mM glucose) according to previously described conditions. To reproduce the pH conditions existing in vivo in the small intestinal mucosa, the apical (AP) solutions were maintained at pH 6.0 (buffered with 10 mM morpholino ethane sulfonic acid), and the basolateral (BL) solutions were maintained at pH 7.4 (buffered with 10 mM *N*-2-hydroxyethylpiperazine-*N*-4-butanesulfonic acid). Prior to transport experiments, cells were washed twice with 500 µL PBS containing Ca^++^ and Mg^++^. Samples transportation by mature Caco-2 cells was assayed by loading the AP compartment with FeSO_4_, FS, and/or labeled FS at 0.5 mg/mL concentration in the AP transport solution (500 µL) and the BL compartment with the BL transport solution (700 µL). The plates were incubated at 37 °C and the BL solutions were collected after 60 min. All BL and AP solutions collected at the end of the transport experiment were stored at −80 °C prior to analysis.

### 2.7. Determination of Trans-Epithelial Transported Iron

#### 2.7.1. Atomic Absorption Spectroscopy Analysis

A Thermo Scientific™ iCE™ 3300 AAS equipped with a Thermo autosampler was used to measure iron in two different ways. In particular, Flame and Zeeman graphite-furnace atomic absorption spectrometer were used respectively, according to iron concentration in different samples. In BL samples, iron concentration was in the range of tens of μg/mL, while, in AP samples, iron concentration was in the range of hundreds of μg/mL. Indeed, Flame analysis was used to quantify iron in AP samples, and the Spectrometer parameters employed were the following: Wavelength 248.3; Band pass 0.2 nm; Background correction D2; Lamp current 75%; Signal Continuous Flame Type Air-Acetylene, Fuel flow rate 0.9 L/min; Measurement time 4 s. Analysis were made in triplicates. Zeeman graphite-furnace atomic absorption spectrometer with pyrolytically coated graphite tubes was used for measuring the iron concentration in the BL sample under the following furnace operating conditions: wavelength used 271.9 nm (Tertiary) for sensitive reduction of three-time, Zeeman Background correction; Lamp current 75%. Analysis was performed in triplicate. For the atomization ramp used during the analysis, the employed conditions were the following: the drying stage graphite tube was warmed for 1 s to 100 °C and held for 30 s; the pyrolysis stage graphite tube was then heated for 20 s to a temperature of 1100 °C and quickly heated to atomization temperatures of 2100 °C, and held for 3 s; cleaning stage was done at 2500 °C for 5 s. Samples were diluted 1:100 (HCl 0.5% and Triton × 100 0.05%).

#### 2.7.2. Fluorescence Quantification of Absorbed Labeled FS

For Labeled FS absorption evaluation, the fluorescence signals were acquired. In detail, 100 μL of collected AP and BL solutions were placed in a black 96-well plate and the fluorescence signals (ex./em. 530/580 nm) were detected using the Synergy H1 fluorescent plate reader from Biotek.

#### 2.7.3. Morphological Studies

After incubation (60 min) in the absence or in the presence of 0.5 mg/mL of FS-MC540 in the AP compartment, cells were washed and fixed with 2% paraformaldehyde in PBS with Ca^++^ and Mg^++^, for 30 min at RT. Filamentous actin (F-actin) was stained with 0.25 µM FITC-conjugated phalloidin and cell nuclei were counter-stained by adding 300 nM DAPI directly to mounting medium (ProLong^®^ Antifade Thermo-fisher Scientific, Milan, Italy). Specimens were analyzed using an inverted laser-scanning confocal microscope equipped with a 40× oil-immersed objective (LSM 700; Carl Zeiss, Germany). Serial optical sections were processed with ZEN 2009 software (Carl Zeiss, Jena, Germany). Images represent single plane optical sections and z-stack elaborations.

### 2.8. Statistical Analysis

Statistical analyses were performed by One-Way ANOVA followed by Dunnett’s and Tukey’s posthoc tests and using GraphPad Prism 9 (San Diego, CA, USA). Values were reported as means ± S.D.; *p*-values < 0.05 were considered to be significant.

## 3. Results

### 3.1. Evaluation of Caco-2 Cells Viability

**Figure 1:**
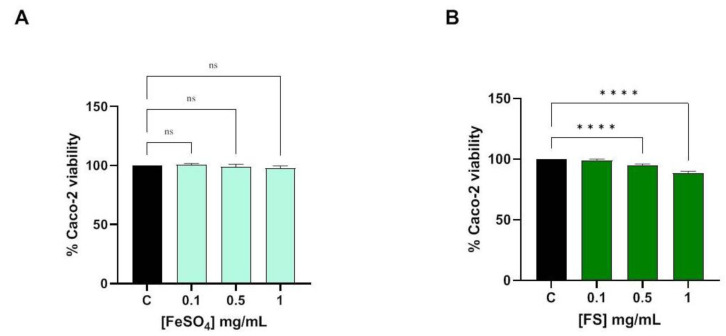
Effect of FeSO_4_ (**A**) and FS (**B**) on the Caco-2 cells viability. Data represents the mean ± s.d. of four independent experiments performed in duplicate. C: untreated cells. (****), *p* < 0.0001; n.s. not significant.

Before proceeding to trans-epithelial transport in Caco-2 cells, the MTT experiments were assessed to verify the possible cytotoxicity exerted by samples. The results suggested that FeSO_4_ is safe at all the tested concentrations (Figure 1A), whereas cellular vitality for FS-treated cells was 98.79 ± 1.33%, 95.00 ± 1.02%, and 88.15 ± 1.94% at 0.1, 0.5 and 1.0 mg/mL, respectively (Figure 1B).

### 3.2. Caco-2 Cell Monolayers Integrity Evaluation after FeSO_4_ and FS Treatment

**Figure 2:**
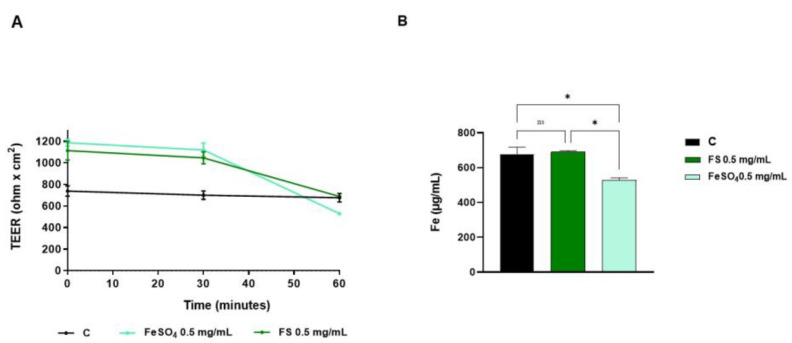
(**A**) Time course of TEER changes recorded in untreated (control), FeSO_4_, and FS-treated Caco-2 cells. (**B**) TEER values after 60 min. Data are the mean ± S.D. of an experiment performed in duplicate. ns: not significant; (*) *p* < 0.05.

As shown in Figure 2A, Control cells displayed a stable TEER baseline throughout all the experimental time, while, in the presence of iron ions, TEER values were higher up to 30 min and reached values comparable to untreated control cells after 60 min treatment. These variations in Fe-treated cells were likely due to ions movements across the monolayers due to different media compositions. However, after 60 min, no significant differences were observed between FS treated and control cells (Figure 2B).

### 3.3. Determination of Trans-Epithelial Transported Iron through Atomic Absorption Spectrometer Analysis

**Figure 3:**
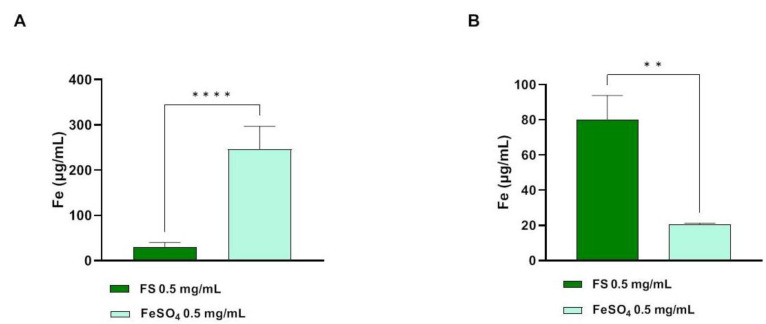
Atomic absorption spectrometric determinations of iron (µg/mL) in AP (**A**) and BL (**B**) compartments, after 60 min treatment with FS and FeSO_4_ at 0.5 mg/mL. Data are the mean ± S.D. of an experiment performed in triplicate. (**) *p* < 0.01; (****), *p <* 0.0001.

**Figure 4:**
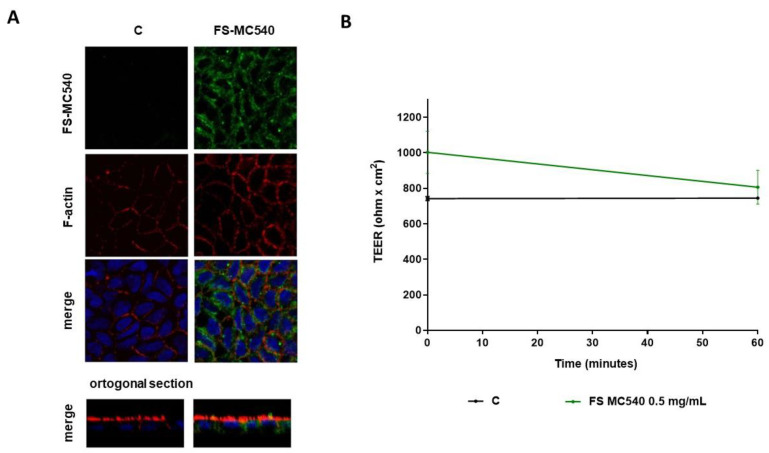
(**A**) Intracellular localization of stained (MC540) FS in polarized Caco-2 cells. FS-MC540: green; nuclei, DAPI: blue; F-actin, FITC-conjugated phalloidin: red. Upper panel Single optical section (x–y) Z-Stacks: a cross-sectional view of z-stack (z–y bottom panel). (**B**) Time course of TEER changes recorded in untreated (control), and FS-MC540 treated Caco-2 cells. Data are the mean ± S.D. of two experiments performed in duplicate.

The atomic absorption spectrometric method (AAS) has been applied for the quantification of the trans-epithelial transported iron across intestinal Caco-2 cells. As shown in Figure 3, after 60 min treatments, iron concentrations in AP compartments (500 µL) were 30.17 ± 10.21 and 246 ± 50.9 µg/mL for FS and FeSO_4_ tested at 0.5 mg/mL, respectively (Figure 3A). In parallel, the collected BL solutions (700 µL) were analyzed in order to determine the amount of trans-epithelial transported iron. Figure 4B clearly shows that, for FS and FeSO_4_ tested at 0.5 mg/mL, the concentrations of total iron were 79.88 ± 13.95 and 20.61 ± 0.67 µg/mL, respectively (Figure 3B).

### 3.4. Fluorescent Evaluation of FS Absorption in Caco-2 Cells 

The evaluation of FS transport across Caco-2 cells was carried out in-depth, labeling the sample with MC540 and measuring the fluorescence of both AP and BL solutions. Table 2 shows that, after 60 min treatment with FS-MC540 at 0.5 mg/mL, the fluorescent signal intensity of the AP (t_60_) solution is highly reduced with respect to the initial fluorescence (AP t_0_), while no fluorescent signal is detectable in the BL (t_60_) solution.

In accordance with fluorescence results, the morphological analysis by confocal microscope detected the intracellular green signals from the cell cytoplasm indicating that, after 60 min treatment, FS can enter into Caco-2 cells (Figure 4A).

In parallel, the measured TEER values were 688.8 ± 7.92 and 677.6 ± 39.6 Ω × cm^2^ for FS-MC540 treated and untreated cells, respectively (Figure 4B).

## 4. Discussion

It is known that the bioavailability of iron salts (FeSO_4_) is low, especially for ferric formulations. Absorption is further impaired when administered at a high dose (by upregulation of hepcidin levels, which remain elevated over 24 h and tend to reduce the absorption of the next oral iron dose) [22] or when co-administered with food or drugs (e.g., antacids, proton pump inhibitors) [23]. As a result, its use is associated with gastrointestinal side effects, due to the interaction of unabsorbed iron with the enterocytes [15,24]. All these may significantly affect compliance with treatment and its efficacy. Therefore, it is quite important to have new iron formulations that show better safety and efficacy than standard ones. In this context, our results clearly demonstrated the safety and the trans-epithelial transported efficacy of Ferro Supremo, a formulation of liposomal iron enriched with vitamins and micronutrients (FS) compared to FeSO_4_ in the presence of vitamin C. To achieve this goal, a differentiated Caco-2 cell has been used as a standard and worldwide preclinical model used for assessing the in vitro bioavailability and safety of different xenobiotics, including food and/or food supplements [25,26] and iron supplements [27]. In particular, preliminary experiments suggested that FS is not able to severely affect the viability of Caco-2 cells measured by MTT. Notably, FeSO_4_ is safe at all the tested concentrations (Figure 1A), whereas FS slightly reduced the Caco-2 viability by 5% and 12% at 0.5 and 1.0 mg/mL (*p* < 0.0001) after 48 h, respectively, whereas it is completely safe at 0.1 mg/mL. MTT assay has been used just as a preliminary test being aware that 48 h of intestinal cell exposition to iron is unrealistic and unphysiological condition. Therefore, these results are not an indication of toxicity but rather an indirect confirmation of the fact that FS is much more able to enter the Caco-2 cells than FeSO_4_. Based on these preliminary results, we decided to assess the qualitative and quantitative ability of FS to be transported by intestinal cells at the fixed concentration of 0.5 mg/mL for 1 h. In particular, it has been well established that Caco-2 cells can undergo spontaneous differentiation in culture conditions and exhibit the characteristics of mature enterocytes [28]. The cell surface facing the top medium develops a brush border that resembles the luminal membrane of the intestinal epithelium. The cell surface attaching to the permeable membrane and facing the bottom medium develops into the basolateral membrane [29]. Despite their colonic origin, Caco-2 cells express the morphological and functional characteristics of small intestinal cells. The Caco-2 monolayer houses multiple transporters, receptors, and metabolic enzymes such as cytochrome P450 1A (CYP1A), sulfotransferases (SULTs), UDP-glucuronosyltransferases (UGTs), and glutathione S-transferases (GSTs) [29].

Trans-epithelial transport experiments were carried out using filter-based inserts and the integrity of the Caco-2 monolayer is controlled by measuring the trans-epithelial electrical resistance (TEER). Briefly, the TEER is a non-invasive technique, that measures the impedance between the lumen and basolateral tissue. TEER measurements use a constant direct current applied by two electrodes, one connected with the lumen side and the other one with the BL side. By applying Ohm’s law, it is possible to measure the related cell resistance [29], which is correlated to the integrity of the differentiated cell monolayer. Indeed, acute toxic effects exerted by xenobiotics may affect the tight junction stability altering the cellular permeability which results in a decrease in TEER values. In light of this consideration, our findings suggested that FS is safe for the cell monolayer integrity and permeability, whereas after 60 min a slight but significant decrease in TEER values was observed for FeSO_4_ (Figure 2B). Our results are in line with the study carried out by Micheletto and colleagues, who investigated the intestinal tolerability of both a standard iron salt formulation and a granular formulation composed of ferric pyrophosphate, modified starch, and phospholipids. Exploiting Caco-2 cells to perform absorption studies, they demonstrated that these types of iron supplements did not induce any alterations in cellular viability and barrier integrity [8]. Based on these results, the apical and basolateral iron amounts were quantified by atomic absorption spectrometric determinations technique after 60 min (Figure 3) and in parallel the qualitative and semiquantitative localization of FS was observed by labelling FS with a fluorescent marker (Figure 4). More in detail, results depicted in Figure 3 demonstrated that, in differentiated Caco-2 cells, iron absorption from FS formulation was greatly enhanced (4 times) as compared to the FeSO_4_ sample (Figure 3B). In agreement with this result, the opposite trend was observed on the apical side, where the concentration of FS is less than FeSO_4_, indicating that FS iron was more efficiently up taken from intestinal cells (eight times) (Figure 3A). This trend agrees with our hypothesis; therefore, qualitative experiments were performed to better characterize the phenomenon. Hence, FS liposomes have been labelled with MC540, and the fluorescence of both AP and BL solutions was measured. Table 2 shows that, after 60 min treatment with FS-MC540 at 0.5 mg/mL, the fluorescent signal intensity of the AP (t_60_) solution is highly reduced with respect to the initial fluorescence (AP t_0_), while no fluorescent signal is detectable in the BL (t_60_) solution. This result suggested that human intestinal differentiated Caco-2 cells were able to uptake and accumulate FS liposome at the intracellular level. Upon loss of liposome structural integrity, the iron is then intracellularly delivered and transported to the BL side. This hypothesis has been confirmed by qualitative confocal microscope analysis. Indeed, Figure 4A clearly shows the intracellular localization of FS-MC540. Compared to our work, differences in the bioavailability of iron present in iron supplements having different compositions and formulations have been previously reported in the literature. Previous studies demonstrated how different formulations can directly affect the overall bioavailability of this micronutrient, by quantifying the absorbed iron in the BL compartment exploiting Caco-2 cells plated in transwell systems [8,30]. Investigating the safety and efficacy of innovative iron formulations represents a fundamental issue to meet the needs of iron deficiency in different population targets, mitigating the liabilities of commonly used iron salts, and improving their performances. In detail, our results are coherent with recent research evidence, supporting that encapsulating vitamins and minerals with liposomes helps to improve overall bioavailability, providing a protective barrier around the compound that helps to shelter the nutrient from degradation and oxidation, besides preserving the digestive tract from potential irritation by the nutrient [31]. The mechanism through which the FS sample is transported by Caco-2 cells will be further investigated but it is reasonable to consider that it might be through phagocytosis much more than paracellular diffusion since we have not observed the decrease in TEER values (Figure 4B) nor detected the fluorescence in the BL side (Table 2).

## 5. Conclusions

In conclusion, our findings clearly suggest that the new FS formulation does not affect the intestinal barrier in in vitro predictive model of Caco-2 cells and that it is four times more efficiently absorbed by Caco-2 cells as compared to FeSO_4_, demonstrating that this formulation can be considered a valuable and efficiently good choice as food supplements for improving the iron deficiency. Further experiments will be carried out to deepen the molecular mechanism through which FS, thanks to the formulation, is better transported at the intestinal level.

## Figures and Tables

**Table 1:** Composition of Ferro Supremo.

Ferro Supremo	%
Iron	6.5
Vitamin C	13
Copper	0.2
Vitamin B2	0.3

**Table 2:** The fluorescent signal intensity of the AP and BL solutions after 60 min treatment with FS-MC540.

Sample	Solution/Time	Fluorescence (RFU)	Fluorescence (%)
FS-MC540	AP t_0_	44,532.0 ± 809.60	100.00 ± 0.00
	AP t_60_	8768.0 ± 508.80	19.69 ± 1.14
	BL t_60_	105.03 ± 10.31	0.24 ± 0.023

## Data Availability

The data used to support the findings of this study can be made available by the corresponding author upon request.

## References

[1] Maladkar M., Sankar S., Yadav A. (2020). A Novel Approach for Iron Deficiency Anaemia with Liposomal Iron: Concept to Clinic. J. Biosci. Med..

[2] Abbaspour N., Hurrell R., Kelishadi R. (2014). Review on Iron and Its Importance for Human Health. J. Res. Med. Sci..

[3] Bresson J.L., Burlingame B., Dean T., Fairweather-Tait S., Heinonen M., Hirsch-Ernst K.I., Mangelsdorf I., McArdle H., Naska A., Neuhäuser-Berthold M. (2015). Scientific Opinion on Dietary Reference Values for Iron. EFSA J..

[4] Zhang A.S., Enns C.A. (2009). Iron Homeostasis: Recently Identified Proteins Provide Insight into Novel Control Mechanisms. J. Biol. Chem..

[5] Hunt J.R., Zito C.A., Johnson L.A.K. (2009). Body Iron Excretion by Healthy Men and Women. Am. J. Clin. Nutr..

[6] Hooda J., Shah A., Zhang L. (2014). Heme, an Essential Nutrient from Dietary Proteins, Critically Impacts Diverse Physiological and Pathological Processes. Nutrients.

[7] Friling M., García-Muñoz A.M., Perrinjaquet-Moccetti T., Victoria-Montesinos D., Pérez-Piñero S., Abellán-Ruiz M.S., Luque-Rubia A.J., García-Guillén A.I., Cánovas F., Ivanir E. (2022). Tolerability of Oral Supplementation with Microencapsulated Ferric Saccharate Compared to Ferrous Sulphate in Healthy Premenopausal Woman: A Crossover, Randomized, Double-Blind Clinical Trial. Int. J. Mol. Sci..

[8] Micheletto M., Gaio E., Tedesco E., Di Maira G., Mantovan E., Zanella M., Pastore P., Roverso M., Favaro G., Benetti F. (2022). Intestinal Absorption Study of a Granular Form of Ferric Pyrophosphate. Metabolites.

[9] Zhao X., Zhang X., Xu T., Luo J., Luo Y., An P. (2022). Comparative Effects between Oral Lactoferrin and Ferrous Sulfate Supplementation on Iron-Deficiency Anemia: A Comprehensive Review and Meta-Analysis of Clinical Trials. Nutrients.

[10] Georgieff M.K., Krebs N.F., Cusick S.E. (2019). The Benefits and Risks of Iron Supplementation in Pregnancy and Childhood. Annu. Rev. Nutr..

[11] DeLoughery T.G. (2017). Iron Deficiency Anemia. Med. Clin. N. Am..

[12] Hurrell R.F. (1997). Preventing Iron Deficiency through Food Fortification. Nutr. Rev..

[13] Christides T., Wray D., McBride R., Fairweather R., Sharp P. (2015). Iron Bioavailability from Commercially Available Iron Supplements. Eur. J. Nutr..

[14] Piskin E., Cianciosi D., Gulec S., Tomas M., Capanoglu E. (2022). Iron Absorption: Factors, Limitations, and Improvement Methods. ACS Omega.

[15] Tolkien Z., Stecher L., Mander A.P., Pereira D.I.A., Powell J.J. (2015). Ferrous Sulfate Supplementation Causes Significant Gastrointestinal Side-Effects in Adults: A Systematic Review and Meta-Analysis. PLoS ONE.

[16] Bloor S.R., Schutte R., Hobson A.R. (2021). Oral Iron Supplementation—Gastrointestinal Side Effects and the Impact on the Gut Microbiota. Microbiol. Res..

[17] (2018). Oral Liposomal Iron: A Treatment Proposal for Anemia. World J. Anemia.

[18] Siddiqui A.S., Ashraf T., Imran U. (2018). Palatability of Micro-Encapsulated Iron Pyrophosphate (Ferfer^®^). Int. J. Clin. Trials.

[19] Donnarumma M., Marasca C., Palma M., Vastarella M., Annunziata M.C., Fabbrocini G. (2020). An Oral Supplementation Based on Myo-Inositol, Folic Acid and Liposomal Magnesium May Act Synergistically with Antibiotic Therapy and Can Improve Metabolic Profile in Patients Affected by Hidradenitis Suppurativa: Our Experience. G. Ital. Dermatol. Venereol..

[20] Yu P.-P., Chang Y.-Z. (2015). Iron Liposome: A More Effective Iron Supplement for Sports Anemia and Anemia of Inflammation. J. Pharm. Care Health Syst..

[21] Ferruzza S., Rossi C., Scarino M.L., Sambuy Y. (2012). A Protocol for Differentiation of Human Intestinal Caco-2 Cells in Asymmetric Serum-Containing Medium. Toxicol. Vitr..

[22] Moretti D., Goede J.S., Zeder C., Jiskra M., Chatzinakou V., Tjalsma H., Melse-Boonstra A., Brittenham G., Swinkels D.W., Zimmermann M.B. (2015). Oral Iron Supplements Increase Hepcidin and Decrease Iron Absorption from Daily or Twice-Daily Doses in Iron-Depleted Young Women. Blood.

[23] (2017). Global, Regional, and National Incidence, Prevalence, and Years Lived with Disability for 328 Diseases and Injuries for 195 Countries, 1990–2016: A Systematic Analysis for the Global Burden of Disease Study 2016. Lancet.

[24] Cancelo-Hidalgo M.J., Castelo-Branco C., Palacios S., Haya-Palazuelos J., Ciria-Recasens M., Manasanch J., Pérez-Edo L. (2013). Tolerability of Different Oral Iron Supplements: A Systematic Review. Curr. Med. Res. Opin..

[25] Awortwe C., Fasinu P.S., Rosenkranz B. (2014). Application of Caco-2 Cell Line in Herb-Drug Interaction Studies: Current Approaches and Challenges. J. Pharm. Pharm. Sci..

[26] Foglieni C., Cavarelli M., Piscopiello M., Fulgenzi A., Ferrero M. (2011). Mn Bioavailability by Polarized Caco-2 Cells: Comparison between Mn Gluconate and Mn Oxyprolinate. Nutr. J..

[27] Scheers N.M., Almgren A.B., Sandberg A.S. (2014). Proposing a Caco-2/HepG2 Cell Model for in Vitro Iron Absorption Studies. J. Nutr. Biochem..

[28] Sambuy Y., De Angelis I., Ranaldi G., Scarino M.L., Stammati A., Zucco F. (2005). The Caco-2 Cell Line as a Model of the Intestinal Barrier: Influence of Cell and Culture-Related Factors on Caco-2 Cell Functional Characteristics. Cell Biol. Toxicol..

[29] Kamiloglu S., Capanoglu E., Grootaert C., van Camp J. (2015). Anthocyanin Absorption and Metabolism by Human Intestinal Caco-2 Cells—A Review. Int. J. Mol. Sci..

[30] Uberti F., Morsanuto V., Ghirlanda S., Molinari C. (2017). Iron Absorption from Three Commercially Available Supplements in Gastrointestinal Cell Lines. Nutrients.

[31] Ko J., Yoo C., Xing D., Gonzalez D.E., Jenkins V., Dickerson B., Leonard M., Nottingham K., Kendra J., Sowinski R. (2023). Pharmacokinetic Analyses of Liposomal and Non-Liposomal Multivitamin/Mineral Formulations. Nutrients.

